# Biosynthesis of Silver Nanoparticles Using Phytochemicals Extracted from Aqueous *Clerodendrum glabrum* for Anti-Diabetes and Anti-Inflammatory Activity: An In Vitro Study

**DOI:** 10.3390/nano15201560

**Published:** 2025-10-14

**Authors:** Kulani Mhlongo, Innocensia Mangoato, Motlalepula Matsabisa

**Affiliations:** African Medicines Innovations and Technologies Development, Department of Pharmacology, School of Clinical Medicine, Faculty of Health Sciences, University of the Free State, Bloemfontein 9301, South Africa; mangoatoi@ufs.ac.za (I.M.); matsabisamg@ufs.ac.za (M.M.)

**Keywords:** *Clerodendrum glabrum*, silver nanoparticles, anti-diabetes, anti-inflammation

## Abstract

This study synthesised silver nanoparticles using an aqueous extract from *Clerodendrum glabrum* and investigated their potential anti-diabetic and anti-inflammatory activity. Diabetes and inflammation are conditions affecting millions worldwide, and the current medications result in side effects. Silver nanoparticles (Ag NPs) were synthesised using *C. glabrum* aqueous extract. Nanoparticles were characterised using ultraviolet–visible (UV–vis) spectroscopy, high-resolution transmission electron microscopy (HR-TEM), and dynamic light scattering (DLS). CG-Ag nanoparticles (CG-Ag NPs) were further evaluated for their nitric oxide (NO) scavenging activity; inhibition of α-amylase, α-glucosidase, and hyaluronidase enzymes; and cytotoxic potential. HR-TEM revealed CG-Ag NPs with an average particle size of 16 nm for 10 mg of plant extract, while 40 mg produced 35 nm, and EDS confirmed the presence of silver elements. The synthesised CG-Ag NPs showed good anti-diabetic and anti-inflammatory activity by inhibiting 93.3% of α-amylase at 6.25 µg/mL, 99.25% of α-glucosidase at 0.95 µg/mL, and 79.6% of hyaluronidase at 100 µg/mL. The NPs also scavenged 96.58% of NO at 250 µg/mL. These results suggest that *C. glabrum* aqueous extract is a green resource for the eco-friendly synthesis of Ag NPs and can potentially be utilised as a therapeutic agent for managing diabetes and inflammation.

## 1. Introduction

Green nanotechnology synthesises nanoparticles using eco-friendly methods that are safe, environmentally friendly, and relatively cheap [1]. Both micro- and macroorganisms have been used to synthesise nanoparticles [2,3]. The advantage of biological synthesis is the production of nanoparticles with lower toxicity [4]. The use of green synthesis eliminates the use of toxic reducing agents, organic solvents, and the generation of hazardous by-products [2,5,6]. Metallic nanoparticles have been exploited in the biomedical field due to their large surface area, which allows the attachment of molecules (drugs, alkaloids, proteins, or dyes) through absorption, binding, or entrapment [7]. Examples of metallic nanoparticles include gold, silver, palladium, platinum, copper, selenium, iron, and their oxides, such as zinc or iron [8]. Silver nanoparticles (Ag NPs) have shown lower toxicity towards mammalian cells and have presented various types of biological activity, such as anti-microbial, anti-cancer, anti-inflammatory, anti-insecticidal, wound healing, antioxidant, hepatoprotective, and anti-diabetic [9,10].

Plant extract synthesis is currently the preferred method because it is cheap, simple (one-vessel reaction), and easily scalable. The phytochemicals in the plant extract, such as terpenoids, phenolics, flavonoids, ketones, amides, aldehydes, alkaloids, and carboxylic acids, facilitate synthesis by acting as reducing and capping agents [11]. Phytochemicals are also responsible for the biological activity of the herbal formulation [1]. Therefore, nanoparticles produced from these extracts have similar activity to the extracts used to synthesise them [1,12,13]. Ag NPs synthesised using *Clerodendrum glabrum E. Mey.* var. *glabrum* aqueous extracts may have similar biological activity to the respective extract.

Using herbal extracts to synthesise metallic nanoparticles will also facilitate the delivery of the phytochemicals and address some common drawbacks of herbal formulations. Herbal medicine’s drawbacks are mainly due to the insolubility of the active constituents, leading to lower bioavailability and increased systemic clearance. Consequently, frequent administration or higher doses are then required to achieve efficacy, rendering the drug a low-class drug for therapeutic use [14]. In phytoformulation, nanotechnology-based systems have shown enhancements in solubility and bioavailability, improvements in stability, the suppression of toxicity, improvements in pharmacological activity, sustained delivery, improved tissue macrophage circulation, and defence against physical and chemical degradation [7,14,15]. For example, a polyethylene glycol nanoformulation showed potential to overcome quercetin’s fast excretion, low water solubility, and low absorption [16].

Inflammation is a biological process activated by the body’s immune system in response to tissue damage or infection [17]. Acute inflammation is required to repair and remove the damaged tissue at the site of infection and usually lasts for a short period [18]. Immune cells (macrophages, dendritic cells, lymphocytes, endothelial cells, fibroblasts, and mast cells) release cytokines and chemokines to facilitate the repair and elimination of the pathogen [19]. However, unregulated and chronic inflammation in conditions such as cancer, asthma, infections, inflammatory bowel disease, diabetes, and rheumatoid arthritis is accompanied by adverse effects. Additionally, inflammation can be responsible for the onset of these diseases [1]. The hyaluronidase enzyme is partly responsible for regulating the inflammatory response, as it breaks down hyaluronic acid into components (hyaluronic degradants) that are pro-inflammatory [20]. The current treatment used in the management of inflammation is characterised by adverse side effects such as gastrointestinal bleeding, vomiting, nausea, constipation, and addiction [17,21].

Diabetes mellitus (DM) is a chronic metabolic disease characterised by persistent high blood glucose levels due to defects in cellular insulin function, secretion, or both in specific tissues. Diabetes causes damage to vital organs such as the liver, pancreas, and kidneys. The International Diabetes Federation estimated that 463 million adults (aged 20–79) were living with diabetes in 2019, and the number is expected to rise to 700 million by 2045 [22]. Chronic hyperglycaemia is linked to macrovascular and microvascular complications. These conditions result in damage or death in extreme situations or when diagnosed in late stages [22,23,24]. Diabetes is currently one of the greatest public health problems and causes of morbidity and death worldwide [24,25]. The inhibition of α-amylase and α-glucosidase is one of the most important solutions. This will limit the conversion of starch into glucose, which causes the rise in glucose levels in the blood. However, the current anti-diabetic oral medications used to achieve this, such as acarbose, α-glycosidase inhibitors, and sulfonylureas, have some adverse effects [25].

Despite advancements in medicine, the management of inflammation and diabetes is hampered by several limitations, some of which are mentioned above. There is growing interest in searching for alternative anti-inflammatory and anti-diabetic agents. Therefore, it is worth investigating the role of traditional medicinal plants with a history of use as analgesics (e.g., *Zingiber officinale* var. *rubrum* [26], *Hamamelis virginiana* [27], and *G. dalenii*) and anti-diabetics (e.g., *Zingiber officinale* var. *rubrum*), in combination with nanoparticles, to discover potent anti-inflammatory and anti-diabetic drugs with few to no side effects. The discovery of better drugs will also facilitate patient compliance, as Sinatra (2010) states [28].

*C. glabrum* is a medium-sized tree that can grow up to 12 m. *C. glabrum* belongs to the Lamiaceae family. It is commonly known as tinderwood in English, moswaapeba in Sotho, munukha-tshilongwe in Venda, and umQoqonga in Zulu [29]. The plant leaves are traditionally used to treat coughs, colds, fever, prolapse, sore throats, wounds, mouth ulcers, and diarrhoea. The roots are used for arthritis and fractured bones. The geographical distribution of *C. glabrum* generally comprises the Bushveld regions of Southern Africa, encompassing areas such as South Africa and Zimbabwe [30]. Conditions such as sore throats and arthritis activate pro-inflammatory pathways as a response, resulting in inflammation. Species within the Clerodendrum genus, such as *C*. *infortunatum* L., *C. glandulosum* Lindl., and *C. colebrookianum* Walp., are traditionally used in India as anti-diabetic medications [31]. Hence, there is an interest in evaluating *C. glabrum*’s anti-diabetic properties.

Particles synthesised using green technology have the potential to be utilised in medical applications due to the absence of toxic chemicals. Several plant extracts have been used to synthesise Ag NPs with potential applications as anti-inflammatory and anti-diabetic. For example, *Azadirachta indica* seed extract increased glucose uptake in yeast and inhibited α-amylase. Moreover, it demonstrated in vivo efficacy by lowering blood sugar levels in mice after 30 days of treatment [32]. Similarly, the leaf extract of *Lonicera japonica* Ag NPs enabled the inhibition of α-glucosidase and α-amylase, with an IC_50_ value of 37.86 µg and 54.56 µg, respectively [33]. Ag NPs derived from an aqueous extract of *Cissus vitiginea* leaves prevented protein denaturation, with an IC_50_ of 270 µg/mL, compared to 321 µg/mL for the extract. Ag NPs from *Xylocarps granatum* bark inhibited albumin denaturation, with an IC_50_ of 270 µg/mL [34], while *Moringa peregrina* Ag NPs displayed powerful NO scavenging potential at concentrations below 100 µg/mL [3].

This study aimed to synthesise Ag NPs using an aqueous extract of *C. glabrum* and evaluate their anti-inflammatory and anti-diabetic properties using in vitro systems. This study’s outcomes will potentially be a starting point for the discovery of novel dual anti-inflammatory and anti-diabetic drugs.

## 2. Materials and Methods

### 2.1. Materials

Silver nitrate (AgNO_3_), α-amylase enzyme, α-glucosidase enzyme, hyaluronidase enzyme, acarbose, ascorbic acid, soluble starch, Griess reagent, hyaluronic acid, p-nitrophenyl glucopyranoside, 3,5-dinitrosalicylic acid (DNS), 2,2′-azobis(2-methylpropionamidine), and 6-hydroxy-2,5,7,8-tetramethylchroman-2-C were obtained from Merck. Munktell filter paper 388 was purchased from Lasec. *C. glabrum* (CG) plant material was sourced from the Tiptop and Random Harvest nurseries in Pretoria and Johannesburg, respectively. Dulbecco’s Modified Eagle’s Medium (DMEM) supplemented with glutamine and phenol red, Roswell Park Memorial Institute (RPMI 1640) medium, Dulbecco’s phosphate-buffered saline (DPBS) (1X), heat-inactivated foetal bovine serum (FBS), trypsin–ethylenediaminetetraacetic acid (EDTA) solution 10X, trypan blue solution, dimethyl sulfoxide (DMSO), and doxorubicin hydrochloride were purchased from Sigma Aldrich (Johannesburg, South Africa). 3-(4,5-Dimethylthiazol-2-yl)-2,5-diphenyltertrazolium bromide (MTT) was purchased from Melford Biolaboratories, Ltd. (Suffolk, UK). Human 293 [HEK-293] (ATCC^®^ CRL-1573™) cells were purchased from the American Type Culture Collection (ATCC, Manassas, VA, USA).

### 2.2. Methods

#### 2.2.1. Extraction of Plant Material

*C. glabrum* was collected, cut into small pieces, and dried at ambient room temperature. The dried material was milled into a powder using a hammer mill (Staalmeester, Hartbeesfontein, South Africa). The milled powder was extracted using boiled water. Then, 100 g of *C. glabrum* was extracted with 500 mL of boiled water under constant shaking at 150 rpm, and the mixture was left under shaking for 24 h at ambient room temperature. The extract was then filtered using Munktell filter paper 388 (Munktell Filter AB, Falun, Sweden) using a vacuum filtration system. The filtrate was concentrated using a rotary evaporator (BUCHI Labortechnik AG, Flawil, Switzerland), with the water bath temperature set at 55 °C and the desired vacuum at 25–30 mbar. The concentrated extract was stored in the fridge for the duration of the study.

#### 2.2.2. Biosynthesis of Silver Nanoparticles

The CG-Ag NPs were synthesised using the *C. glabrum* aqueous extract as follows: 10 mL of AgNO_3_ (3 mM) was mixed with 1 mL plant extract (10 mg/mL) at ambient room temperature for 3 h. Before the start of synthesis, the pH of the mixture was checked with a pH meter. The formation of the particles was visually monitored. The absorbance of the solution was also monitored every 1 h for the appearance of a peak between 400 nm and 500 nm using a spectrophotometer. The CG-Ag NPs were purified by centrifuging at 4000 rpm for 30 min, and the pellet was washed with distilled water thrice to remove unused silver ions and phytochemicals. The formed pellet (nanoparticle) was resuspended in 3 mL of distilled water and stored in the fridge until required for use. The formed nanoparticles were confirmed using ultraviolet–visible (UV–vis) spectroscopy (Multiscan Go ascent plate reader, Thermo Scientific, Waltham, MA, USA) at 400–500 nm. The nanoparticle synthesis was optimised by evaluating different parameters, such as the time (30 min, 1 h, 2 h, 3 h, and 24 h), temperature (room temperature, 37 °C, 50 °C, and 70 °C), plant extract concentration (5, 10, 15, 20, 30, and 40 mg/mL), and AgNO_3_ concentration (1 mM,3 mM, 6 mM, 9 mM, and 10 mM). The AgNO_3_ concentration was kept at 3 mM for the evaluation of the other parameters and was only changed when the AgNO_3_ concentration effect was evaluated.

#### 2.2.3. Stability Testing of CG-Ag Nanoparticles

The stability of the CG-Ag NPs in different solutions, namely phosphate-buffered saline (PBS) pH 7.4, Tris (hydroxymethyl)aminomethane hydrochloride (Tris-HCl) buffer pH 7, borate buffer pH 8, RPMI 1640, distilled water (dH_2_O), and DMEM, was determined. In a glass vial, 200 µL of washed CG-Ag NPs was mixed with 1 mL solutions and incubated at 37 °C for 24 h. The stability of the particles was evaluated over a pH range of 2–12 and at different storage temperatures (bench, fridge, 37 °C, 50 °C). The stability of the particles was monitored using UV–vis spectra (Multiscan Go ascent plate reader, Thermo Scientific, Waltham, MA, USA).

#### 2.2.4. Characterisation of Silver Nanoparticles

The formation of CG-AgNPs was confirmed by observing their surface plasmon resonance (SPR) peaks using UV–visible spectroscopy (Multiskan GO spectrophotometer, Thermo Scientific, Waltham, MA, USA) at a wavelength range of 250 to 800 nm. Dynamic light scattering (DLS) was performed using a Zetasizer Nano ZS90 (Malvern, Worcestershire, UK) to determine the particle size distribution, zeta potential, and polydispersity index (PDI) values of the synthesised CG-Ag NPs. High-resolution transmission electron microscopy (HR-TEM) analyses were conducted using a JOEL JEM-F200 multi-purpose electron microscope (Tokyo, Japan) to determine the morphology and average particle size distribution. The particle size distribution was determined using the Image J software, version 1.54p. Chemical analysis was conducted using energy-dispersive X-ray spectroscopy (EDS), (Tokyo, Japan) to confirm the presence of Ag on the formed NPs. HR-TEM and EDS analyses were performed at the Centre for Microscopy at the University of the Free State.

#### 2.2.5. Phytochemical Analyses

Qualitative phytochemical analyses were performed to assess the phytochemicals in *C. glabrum* extract that participate in the formation and stabilisation of silver nanoparticles. The extract and the nanoparticles were qualitatively screened for the presence or absence of tannins, saponins, alkaloids, steroids, anthraquinones, terpenoids, and glycosides based on the adaptation and modification of the methods of Rajkumar et al. [35]. The results are tabulated with negative (−) indicating absence and positive (+) indicating presence. Below is a brief description of the methodology used.

##### Analysing for Flavonoids

An alkaline test was used to screen for flavonoids. Briefly, 2 mL of a filtered sample (plant extract or nanoparticles) was combined with two drops of 20% NaOH. The development of a yellow colour was observed. This was followed by the addition of 3 drops of 70% HCL, and the colour disappeared. The formation and disappearance of the colour indicates the presence of flavonoids.

##### Analysing for Phenols

A ferric chloride assay was used, whereby 2 mL of a filtered sample was mixed with 2 mL of 5% aqueous FeCl_3_. After mixing, the development of a blue–green colour indicates the presence of phenols.

##### Analysing for Tannins

A ferric chloride assay was used, where 2 mL of a filtered sample was mixed with 10% of alcoholic FeCl_3_ (dissolved in ethanol). The development of a black or brownish colour was considered to indicate tannins.

##### Analysing for Alkaloids

Dragendorff reagent was used to test for the presence of alkaloids. Briefly, 1 mL of the sample was dissolved in 1 mL of dilute hydrochloric acid and filtered. Thereafter, 1 mL of Dragendroff’s reagent was added. The resulting red–orange precipitate indicated the presence of alkaloids.

##### Analysing for Terpenoids

A chloroform test was used to assess the presence of terpenoids, where 2 mL of a filtered sample was combined with 0.5 mL chloroform, 0.5 mL of acetic anhydride, and a few drops (2–3) of undiluted sulfuric acid. The development of a reddish brown precipitate indicated the presence of terpenoids.

##### Analysing for Anthraquinones

Briefly, 2 mL of the sample was mixed with 5 mL of chloroform, followed by shaking for 5 min. After 5 min, the mixture was filtered and mixed with 2 mL of 10% ammonia. The development of a pink colour indicated the presence of anthraquinones.

##### Analysing for Saponin

A frothing test was used to detect saponins. Briefly, 2 mL of the sample was mixed with 4 mL of distilled water. The solution was vigorously shaken, and, if the formed froth was stable for more than 10 min, it was considered to indicate saponins.

##### Analysing for Glycosides

A Keller-Killian test was used to assess the presence of glycosides; 2 mL of the sample was mixed with 3 drops of FeCl_3_, 0.5 mL of glacial acetic acid, and 0.5 mL of sulphuric acid. The development of a brown ring between the layers indicated the presence of glycosides.

##### Analysing for Steroids

This was carried out via Salkowski’s test. About 2 mL of the sample was mixed with 2 mL of chloroform. Thereafter, 2 mL of sulphuric acid was added. The formation of a red to brownish layer on the chloroform layer indicated the presence of steroids.

#### 2.2.6. Cytotoxicity of the Extract

Stock solutions of 1 mg/mL of the Ag NPs and *C. glabrum* aqueous extract were prepared by dissolving 1 mg of *C. glabrum* extract in 1 mL dH_2_O. Each stock solution was sterilised by filtration using a 0.2 µm sterile membrane filter (Sigma Aldrich, Johannesburg, South Africa). Human 293 [HEK-293] (ATCC^®^ CRL-1573™) (ATCC, Manassas, VA, USA). cells were cultured and maintained in DMEM, supplemented with 10% FBS. Cells were cultured at 37 °C in 5% CO_2_ in a humidified atmosphere incubator until cells were ready to be subcultured. The cytotoxicity of the test samples was measured using the 3-(4,5-dimethylthiazol-2-yl)-2,5-diphenyltertrazolium bromide (MTT) assay, as previously described by Mosmann (1983) [36]. Briefly, 100 μL/well of a cell suspension (10,000 cells/mL) was seeded in a 96-well microtiter plate and incubated at 37 °C for 24 h. Thereafter, the cells were treated with the test samples by adding 100 μL/well of each concentration in triplicate to obtain a final concentration of 400 µg/mL, 300 µg/mL, or 200 µg/mL for the extracts and 10 µg/mL, 5 µg/mL, 4 µg/mL, 3 µg/mL, 2 µg/mL, or 1 µg/mL for doxorubicin (Sigma Aldrich, Johannesburg, South Africa). The treated plates were further incubated for a period of 72 h.

Following the 72 h treatment incubation period, 100 µL of MTT solution was added to all wells. The plate was left to incubate for 3 h at 37 °C in a CO_2_ incubator. DMSO was added to each well to dissolve the formazan crystals, resulting in a purple solution. The absorbance of each plate was measured using a microplate reader (Multiskan GO ascent plate reader, Thermo Fisher Scientific, Waltham, MA, USA) at a wavelength of 570 nm. The assay was carried out thrice in triplicate. Dose–response graphs were generated using Excel, while the half-maximal inhibitory concentration (IC_50_) values were calculated using the AAT Bioquest (https://www.aatbio.com/tools/ic50-calculator, accessed on 1 August 2025) online tool, indicating the concentrations of the test samples required to inhibit 50% of the cancer cells’ growth relative to the control [37]. The growth inhibition percentage was calculated using the formula below [30]:

% cell growth inhibition = 1 − ((At − Ab)/(Ac − Ab)) × 100

where At = absorbance value of the test compound, Ab = absorbance value of the blank, and Ac = absorbance value of the control.

#### 2.2.7. Investigation of Anti-Diabetic Activity

The anti-diabetic properties of the extract and NPs were investigated using α-glucosidase and α-amylase inhibition assays.

##### α-Glucosidase Activity Inhibition Assay

A spectrophotometric-based method was used to assess the ability of CG-Ag NPs and *C. glabrum* extract to inhibit the α-glucosidase enzyme, adapted from Shayegan et al. [38]. A 1 U/mL α-glucosidase enzyme solution was prepared from 20 U/mg reagent purchased from Sigma-Aldrich in a 50 mM pH 6.8 potassium phosphate buffer. Thereafter, 20 µL of the enzyme was added to a 96-well plate with 135 µL phosphate buffer and 20 µL of plant extract/CG-Ag NPs at different concentrations. The plant extract and acarbose were evaluated at concentrations between 31.25 and 500 µg/mL, while the Ag NPs were assessed between 0.06 and 0.95 µg/mL due to their saturation at sample concentrations of 1 µg/mL and above. The mixture was incubated at 37 °C for 10 min. Thereafter, 25 µL of 4 mM p-nitrophenyl glucopyranoside (substrate) was added to the solution. The solution was further incubated for 20 min at 37 °C. The change in absorbance was measured using a Thermo Scientific Multiskan GO spectrophotometer (Waltham, MA, USA) at 405 nm. Acarbose was used as a positive control, and a buffer was used as a blank (100% activity). Inhibition was calculated using the following formula:

Inhibition = ((A0 − A1)/A0) × 100%

A0 is the absorbance of the control (100% without extract/NPs), and A1 is the absorbance in the presence of the extract.

##### α-Amylase Activity Inhibition Assay

The α-amylase enzyme inhibition assay was conducted using the DNS factory protocol [39]. The *C. glabrum* extracts, CG-Ag 10 NPs, CG-Ag 40 NPs, and acarbose were prepared by dissolving or resuspending them in a phosphate buffer at pH 6.9. The plant extract and acarbose were evaluated at concentrations between 31.25 and 500 µg/mL. In contrast, Ag NPs were assessed at 0.06 and 6.25 µg/mL due to sample saturation at concentrations above these.

Here, 200 µL of α-amylase enzyme was added to clean glass test tubes containing 200 µL of the extract/NPs/acarbose. The mixtures were incubated at 37 °C for 10 min in a water bath. Then, 200 µL of 1% starch was subsequently added to the mixture and it was incubated for 15 min. The reaction was terminated with the addition of 200 µL DNS reagent, followed by heating in an 85 °C water bath for 10 min. The solution was allowed to cool to ambient room temperature and diluted with 2 mL of distilled water. The absorbance was read at 540 nm using a Thermo Scientific Multiskan GO spectrophotometer. The percentage of inhibition was calculated using the equation below. Acarbose and phosphate buffer were used as positive and negative controls, respectively.

Inhibition = ((A0 − A1)/A0) × 100%

A0 is the absorbance of the control (100% without extract/NPs), and A1 is the absorbance in the presence of the extract.

#### 2.2.8. Investigating Anti-Inflammatory Activity

The anti-inflammatory properties of the biogenic NPs and *C. glabrum* extract were evaluated by assessing their ability to inhibit hyaluronidase enzyme activity and their nitric oxide (NO) scavenging abilities.

##### Hyaluronidase Activity Inhibition Assay

The hyaluronidase assay was conducted using a method modified from Bahta et al. [40]. Briefly, 50 µL of 0.01% BSA was added to a 96-well plate, followed by 50 µL of an 800 µg/mL concentration of NPs/extract/indomethacin. Microdilutions were performed to achieve the desired concentration range of 200 µg/mL to 12.5 µg/mL. After adding 50 µL of 5 U hyaluronidase enzyme prepared in 20 mM sodium phosphate buffer (pH 7.0) containing 77 mM sodium chloride, the solution was incubated in an incubator at 37 °C for 15 min. Following incubation, 100 µL of 0.03% hyaluronic acid was mixed in 300 mm sodium phosphate (pH 5.35) and incubated for 45 min at 37 °C. The undegraded hyaluronic acid was precipitated with 1 mL 0.1% BSA solution in 79 mM acetic acid and 24 mM sodium acetate for 10 min at room temperature. The turbidity of the solution was measured at 600 nm. The hyaluronic acid solution with no enzyme was considered 100%. Sample blanks were prepared with different concentrations. The enzyme activity was checked by running a reaction with 0.01% BSA. Indomethacin (Sigma Aldrich, South Africa) was used as a reference anti-inflammatory drug at a concentration of 12.5–200 µg/mL. The degradation inhibition was calculated using the formula below:

Inhibition = (A1 − A0)/(A2 − A3) × 100%

A1 is the absorbance in the presence of the extract, and A0 is the absorbance in the presence of the extract (varying concentration, no hyaluronic acid) and buffer. A2 is the absorbance of the control (100% without extract/NPs), and A3 is the absorbance in the presence of buffers only.

##### Nitric Oxide (NO) Scavenging Assay

The assay was conducted using a method adapted from Kumar et al. [41]. The NO measured in the assay is generated from the spontaneous decomposition of sodium nitroprusside dissolved in 20 mM phosphate buffer (pH 7.4) to a final concentration of 20 mM. The NO interacts with the available oxygen to form nitrate, which is measured by the Griess reaction using a spectrophotometer at a wavelength of 540 nm [42]. Sodium nitroprusside (20 mM, 50 µL) in PBS and 50 µL of plant extract/NPs at different concentrations (12.5 µg/mL to 200 µg/mL) were used. The mixture was incubated for 30 min at ambient room temperature. After 30 min, 125 µL of Griess reagent was added and the mixture was further incubated for 10 min to fully develop the colour. Ascorbic acid (12.5 µg/mL to 200 µg/mL) was used as a standard drug. Plant extract/NP blanks were subtracted from the experiment to account for absorbance due to plant extracts/NP. The experiments were conducted in triplicate. The colour was then measured at 540 nm. The results were calculated using the equation below:

Inhibition = (A0 − A1) − (A3 − A4)/(A0 − A1) × 100%

A0 is the absorbance of the control (100% without extract/NPs), A1 is the absorbance in the presence of buffer only, A3 is the absorbance in the presence of the extract, and A4 is the absorbance in the presence of the extract (varying concentrations) and buffer.

## 3. Results

### 3.1. Synthesis, Optimisation, and Characterisation of the Synthesised CG-Ag NPs

**Figure 1:**
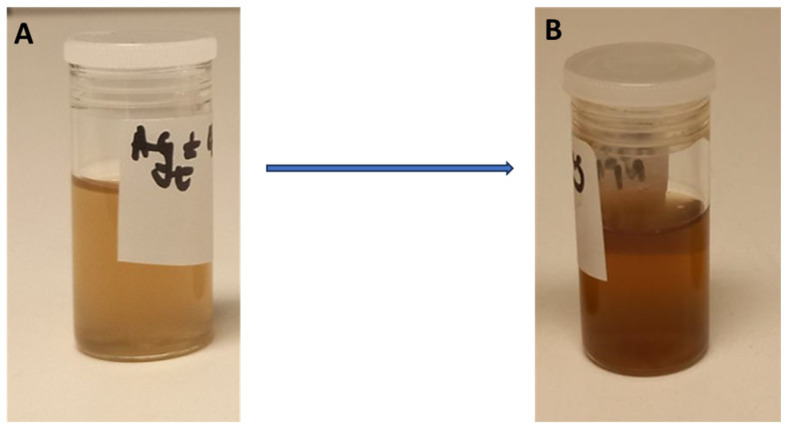
Images of a solution before the synthesis of the *Clerodendrum glabrum*–Ag NPs (**A**) and after the synthesis of the *Clerodendrum glabrum*–Ag NPs (**B**).

**Figure 2:**
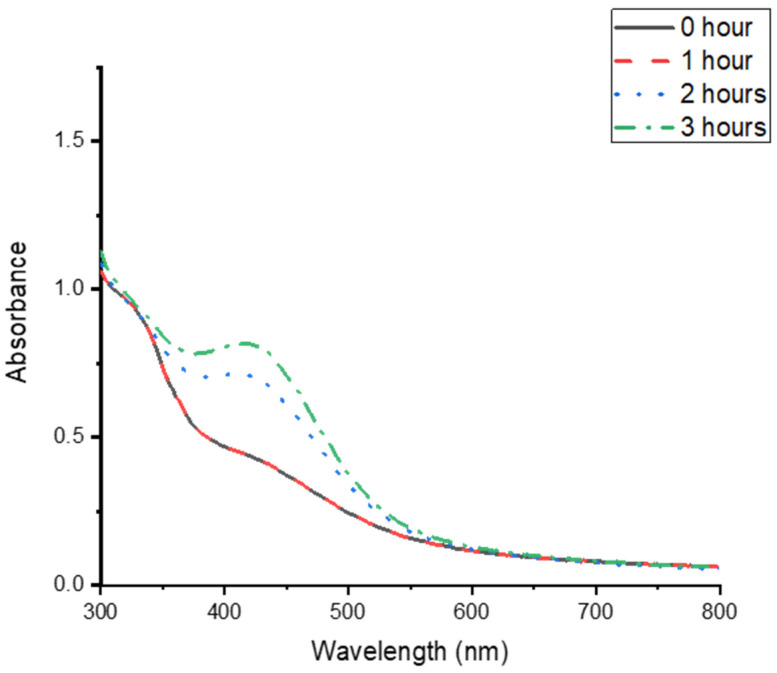
UV–vis absorption spectra of *Clerodendrum glabrum*–Ag NPs synthesised using a 3 mM AgNO_3_ solution and 10 mg/mL *C. glabrum* aqueous extract, monitored at different intervals. The spectra were scanned from 300 nm to 800 nm at 25 °C, and 1 mL of the sample was used to monitor the synthesis progress.

Metallic nanoparticles are formed during the reduction of metal ions. In the case of Ag NPs, the silver ions are converted to Ag [1]. The plant phytochemicals act as reducing agents in forming Ag NPs. The pH of the mixture before synthesis was 6.60. The synthesis of the CG-Ag NPs was confirmed by the colour change of the solution after the incubation period. As illustrated in Figure 1A, the mixture was almost colourless; however, after incubation with *C. glabrum* aqueous extract, the solution changed to an intense brown (Figure 1B), indicating the successful formation of CG-Ag NPs [43]. A yellowish brown colour characterises Ag NPs as a result of their surface plasmon resonance (SPR) vibrations [1,44]. The synthesised particles were confirmed by measuring the UV–vis spectra between 250 and 800 nm (Figure 2). The solution initially showed sharper peaks below 400 nm due to the presence of phytochemicals, with no peaks after 400 nm. However, after 2 h of synthesis, a sharper peak started appearing, as seen in Figure 2, indicating Ag NP formation. The CG-Ag NPs had a maximum absorbance value of 428 nm (Figure 2), a well-known characteristic of Ag NPs. A lower SPR is indicative of smaller particles [1,45,46]. The rate at which particles are formed varies from extract to extract. A rhizome extract of *Alpinia calcarata* showed particle formation in 5 min, while a *Moringa peregrina* extract took 2 h to start showing a colour change, similarly to our extract [3].

### 3.2. Effects of Temperature and Time on the Synthesis of CG-Ag NPs

**Figure 3:**
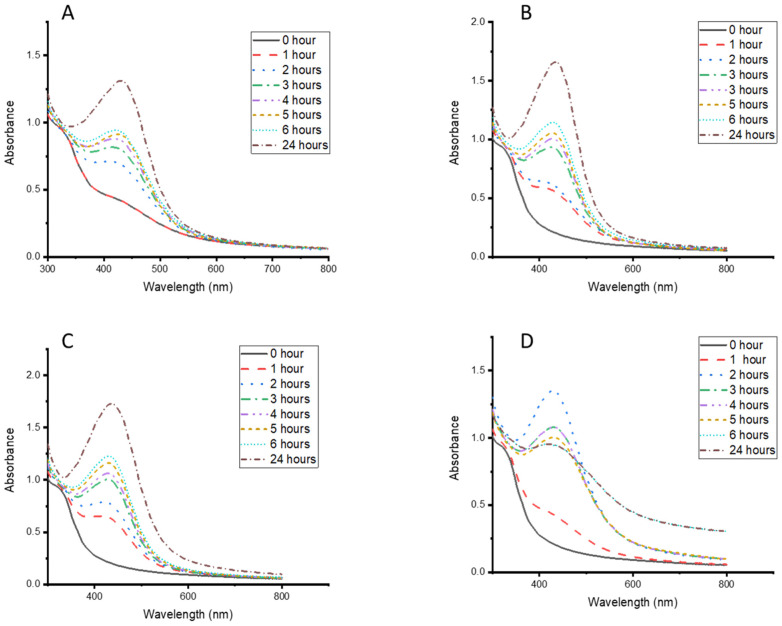
UV–vis absorption spectra of *Clerodendrum glabrum*–Ag NPs synthesised at different temperatures—(**A**) room temperature, (**B**) 37 °C, (**C**) 50 °C, and (**D**) 70 °C—and monitored at various time intervals.

This study evaluated different plant concentrations, temperatures, times, and AgNO_3_ concentrations to obtain the optimum synthesis conditions for the CG-Ag nanoparticles. Figure 3D shows that 70 °C produced CG-Ag NPs with sharper absorbance within two hours. However, after two hours, there was a decrease in absorbance, which could have been due to the degradation or agglomeration of the particles. Additionally, precipitation was observed after 2 h. At ambient room temperature (Figure 3A), the NPs formed more slowly compared to 37 °C, 50 °C, and 70 °C. Based on the above results, particles were synthesised at 70 °C for 2 h as the optimum temperature condition. In addition to an increased reaction rate, higher temperatures are known to produce smaller nanoparticles compared to lower temperatures [47], suggesting that the temperature is an important parameter to consider, depending on the size and application requirements.

### 3.3. Effects of AgNO_3_ Concentration on the Synthesis of CG-Ag NPs

**Figure 4:**
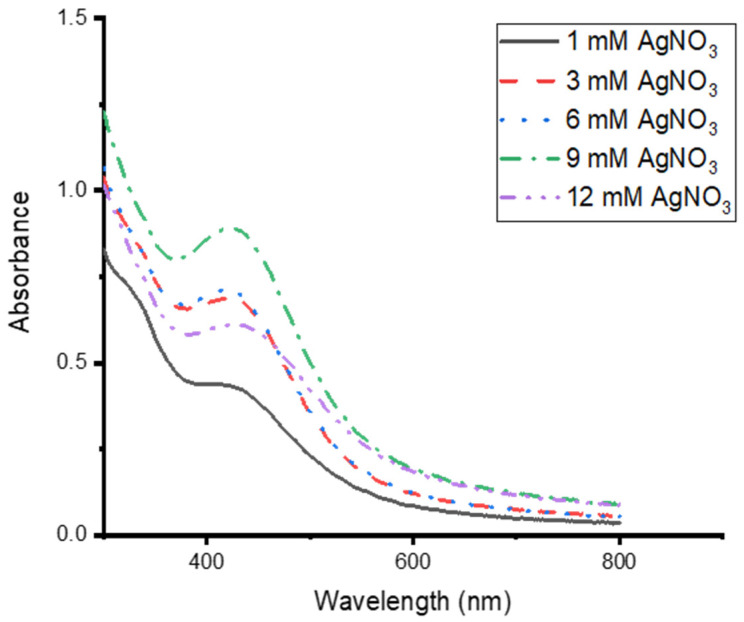
UV–vis absorption spectra of *Clerodendrum glabrum*–Ag NPs synthesised at different AgNO_3_ concentrations (1 mM to 12 mM).

Different concentrations of AgNO_3_ were tested at 70 °C to synthesise CG-Ag NPs, and 9 mM produced the highest concentration of Ag NPs (Figure 4). However, there was the formation of a precipitate in the solution, indicating particle aggregation or larger particle formation. There was a drop in absorbance at a 12 mM AgNO_3_ concentration. The optimum concentrations were 3 mM and 6 mM. It was observed that higher AgNO_3_ concentrations resulted in larger particles. Tyavambiza and co-workers also obtained the optimum synthesis of Ag NPs at 3 mM [1]. A higher concentration of AgNO_3_ is known to produce larger particles. The Ag NP size increased from 32.7 nm to 39.9 nm with an increase in AgNO_3_ from 0.5 to 0.9 mM [48]. A 3 mM AgNO_3_ concentration was chosen for further synthesis, as we were targeting smaller particles, and there was no clear difference between 3 mM and 6 mM.

### 3.4. Effects of CG Extract Concentration on the Synthesis of CG-Ag NPs

**Figure 5:**
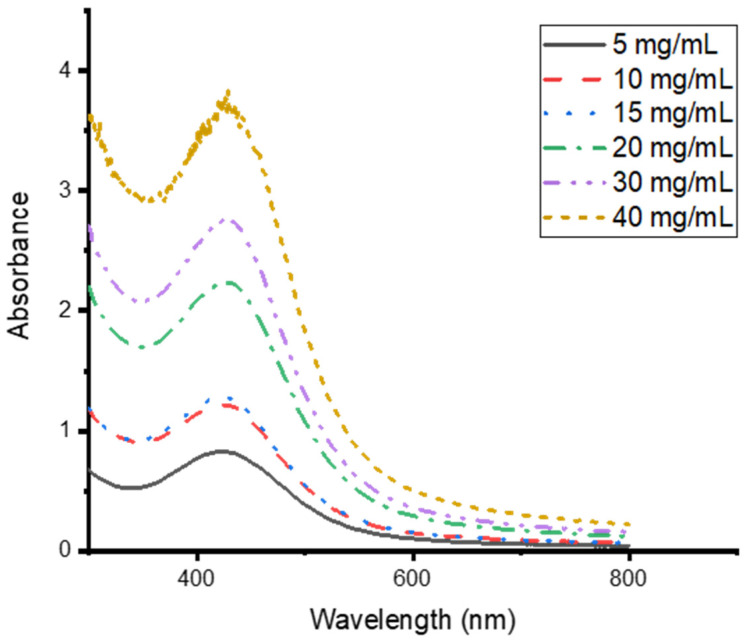
UV–vis absorption spectra of *Clerodendrum glabrum*–Ag NPs synthesised at different *C. glabrum* aqueous concentrations.

It is worth noting that plant phytochemical concentrations differ from plant to plant and within the same plant depending on the season, age, and geological location. Therefore, the reducing power of plant extracts will differ, necessitating the optimisation of the process for individual plant extracts [40]. Different concentrations of the extract were tested for the optimum synthesis of silver nanoparticles, and it was discovered that the higher the concentration of the extract, the greater the absorbance intensity, which correlates with the nanoparticle concentration. This was based on the increase in the absorbance intensity as the plant concentration increased (Figure 5). The spectrum of the nanoparticles synthesised at 40 mg/mL showed an irregular pattern. This could have been a result of detector saturation, as there were high concentrations of NPs and phytochemicals. To confirm this, a smoother spectrum was achieved when the sample was washed and diluted.

It is well established that the absorbance intensity is directly related to the concentration of the nanoparticles [1,49]. The increase in the concentration of the nanoparticles as the extract concentration increases is due to an increase in phytochemicals. This increases the reducing ability of the extract to convert the silver ions into Ag nanoparticles, stabilised and capped with phytochemicals. Similar phenomena were observed by Tyavambiza and co-workers when synthesising silver nanoparticles from *Cotyledon orbiculate* aqueous extract [1]. The synthesis of Ag nanoparticles with *Capparis spinosa* L. leaf extract showed an increase in absorbance as the volume of the extract used in their synthesis increased [50]. It is important to optimise the extract concentration as a lower concentration leads to the insufficient reduction of the Ag ions, while a higher concentration leads to a higher formation rate, which could lead to cluster formation and agglomeration [51].

### 3.5. Stability of the CG-Ag NPs Under Different Conditions

**Figure 6:**
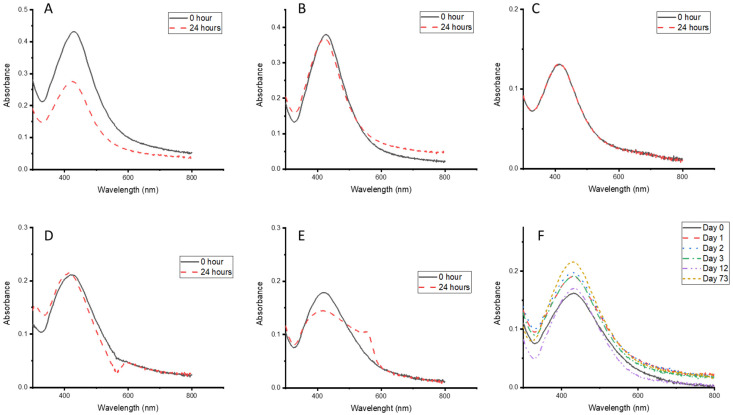
UV–vis absorption spectra of *Clerodendrum glabrum*–Ag NPs stored in different media and buffers: (**A**) Tris-HCI buffer pH 7, (**B**) borate buffer pH 8, (**C**) PBS pH 7.4, (**D**) RPMI, (**E**) DMEM, and (**F**) long-term storage.

Based on the fact that nanoparticles will be stored and tested using different buffers for biomedical uses, their stability in these buffers is of paramount importance. The stability of the particles was evaluated for Tris-HCI (Figure 6A), borate (Figure 6B), PBS (Figure 6C), RPMI (Figure 6D), and DMEM (Figure 6E), and the absorbance was monitored for 24 h. The absorbance of the particles in Tris-HCI (Figure 6A) and DMEM slightly decreased after 24 h, indicating possible particle aggregation, but at a slower rate. The particles remained stable in all other buffers after 24 h, making them suitable media for testing the biological activity of the synthesised CG-Ag NPs, with possible aggregation during the testing period. The synthesised CG-Ag NPs were also stable in an aqueous solution for 73 days when stored on the bench top. Formulation based on this can be helpful in areas where refrigeration is challenging.

**Figure 7:**
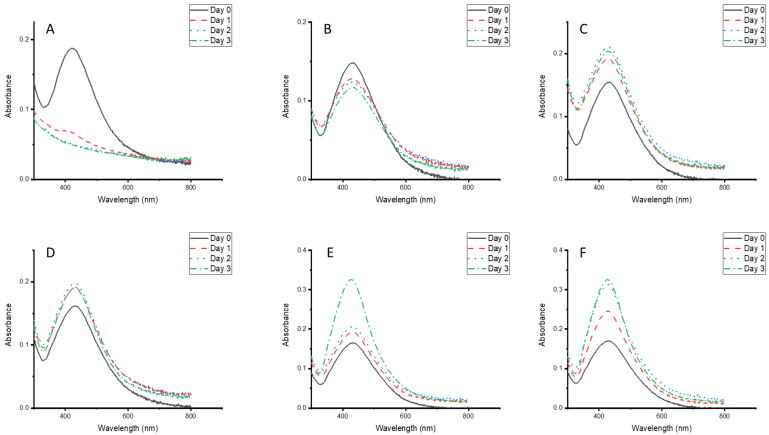
UV–vis absorption spectra of *Clerodendrum glabrum*–Ag NPs stored at different pH levels: (**A**) pH 2, (**B**) pH 4, (**C**) pH 6, (**D**) pH 8, (**E**) pH 10, and (**F**) pH 12.

The CG-Ag particles showed stability at pH 4 to 12 for 3 days, but not at pH 2, where they degraded within 24 h (Figure 7A). Their stability under different pH conditions makes them suitable for storage and application in various formulations at different pHs.

**Figure 8:**
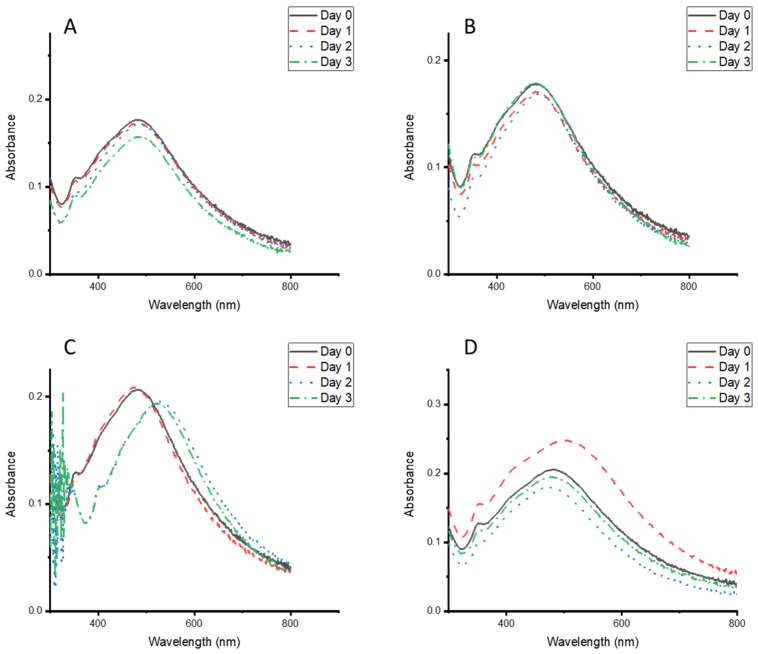
UV–vis absorption spectra of *Clerodendrum glabrum*–Ag NPs stored at different temperatures: (**A**) fridge, (**B**) bench top, (**C**) 37 °C, and (**D**) 50 °C.

When the particle stability was evaluated under different temperature conditions, a red shift occurred at 37 °C after 24 h of storage, and they were stabilised for the next 48 h (Figure 8C). The particles were stable after 72 h of storage under different temperature conditions (Figure 8A–D). These results highlight the suitability of the particles to be stored and used at different temperatures, making them valuable for use in remote areas.

### 3.6. Phytochemical Analysis

Phytochemical analyses of both the synthesised CG-Ag NPs and *C. glabrum* aqueous extracts were performed to evaluate the classes of phytochemicals involved in synthesising the NPs. The phytochemical constituents detected in both the NPs and plant extract included terpenoids, steroids, and saponins, indicating their involvement in the biosynthesis of the particles (Table 1). A *Cucumis prophetarum* aqueous leaf extract yielded similar results to our screening, with the study detecting alkaloids and not screening for steroids, glycosides, or anthraquinones. The study successfully synthesised Ag NPs with a size range between 30 and 50 nm [52]. A study by Matotoka and co-workers detected the presence of tannins, phenols, proanthocyanidins, and flavonoids in a *C. glabrum* aqueous extract, which is in agreement with our study [53]. Further analysis, such as liquid chromatography–mass spectrometry, is required to identify and elucidate the bioactive compounds participating in the synthesis and capping of the Ag NPs.

### 3.7. Dynamic Light Scattering Analysis

The synthesised nanoparticles had zeta potential averages of −25 mV and −21 mV for CG-Ag 10 and CG-Ag 40 NPs, respectively, indicating the successful production of stable CG-Ag NPs (Table 2). A zeta potential above +30 mV or below −30 mV is considered to indicate stability [54,55]. A negative zeta potential shows strongly stable nanoparticles [56]. The synthesised particles were monodispersed as the polydispersity index (PI) was 0.3088 and 0.2687 for CG-Ag 10 and CG-Ag 40 NPs, respectively. A PI value of ≤0.5 is considered to indicate monodispersity [57]. Dynamic light scattering measures the hydrodynamic diameter of the particles and takes into account the phytochemical layer surrounding the particles [1]. Here, the diameter was larger than that obtained from the HR-TEM analysis (Section 3.8). Xu et al. (2023) obtained results whereby the DLS diameter was larger than the TEM diameter for Ag NPs biosynthesised from *Solanum tuberosum* peel aqueous extract [57].

### 3.8. High-Resolution Transmission Electron Microscopy (HR-TEM) Analysis

**Figure 9:**
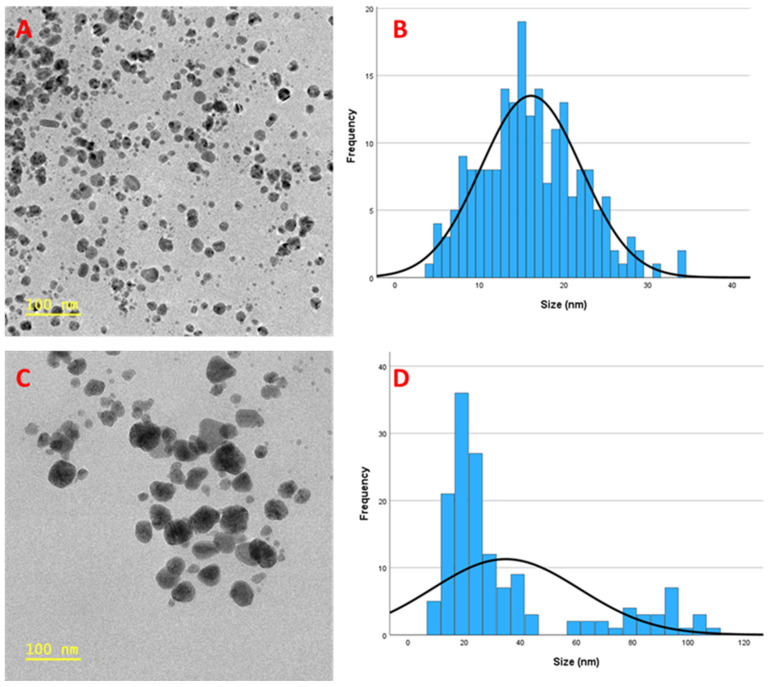
High-resolution transmission electron microscopy analysis of *Clerodendrum glabrum*–Ag NPs at 100 nm scale: CG-Ag 10 (**A**,**B**) and CG-Ag 40 (**C**,**D**). (**A**,**C**) represent TEM images, while (**B**,**D**) represent size distribution histograms.

HR-TEM was used to analyse the morphologies and the sizes of the particles. The analysis revealed that most synthesised particles were relatively spherical (Figure 9A,C) in shape and had an average size of 16 nm and 35 nm for CG-Ag 10 and CG-Ag 40 NPs, respectively, when measured using the Image J software version 1.54g. Figure 9B,D are histograms representing the particle size distribution. Based on the DLS and HR-TEM analyses, we observed that, as the concentration of the extracts increased, so did the average size of the particles. The particles synthesised with 10 mg of the extracts were smaller than those synthesised with 40 mg, suggesting that a lower concentration of plant extracts produces smaller particles. This phenomenon was also observed by Wasilewska and colleagues when synthesising Ag NPs using apple extracts [58].

**Figure 10:**
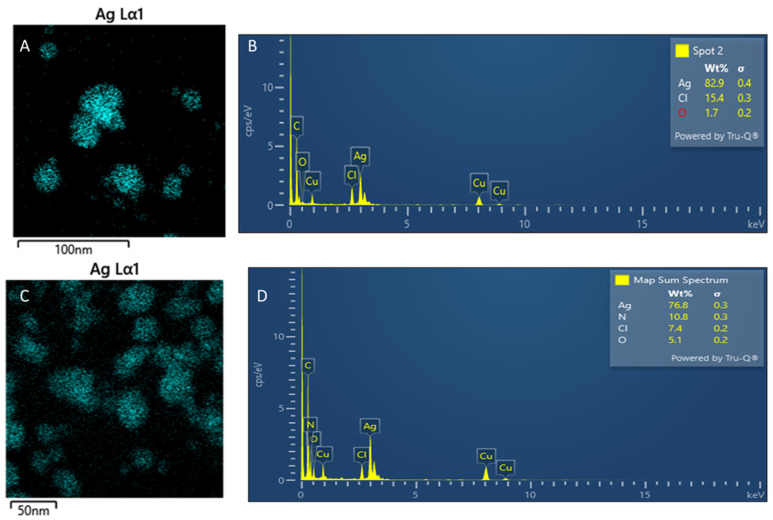
EDS spectrum analysis of the synthesised *Clerodendrum glabrum*–Ag NPs. CG-Ag 10 NPs (**A**,**C**); CG-Ag 40 NPs (**B**,**D**).

EDS was used to analyse the synthesised particles’ elemental compositions to confirm the presence of Ag particles. An absorptive peak around 3 keV indicates the presence of Ag (Figure 10B,D). In contrast, the detection of carbon, oxygen, nitrogen, and chloride indicates the presence of phytochemicals as capping agents for the Ag nanoparticles (Figure 10). These elements are fundamental in phytochemicals. The copper peak is due to the grid used in TEM analysis. Nitrogen was only visible on the CG-Ag 40 particles due to the increased concentrations of phytochemicals. The mapping (Figure 10A,C) confirms that the particles are mainly composed of Ag.

### 3.9. Cytotoxicity

**Figure 11:**
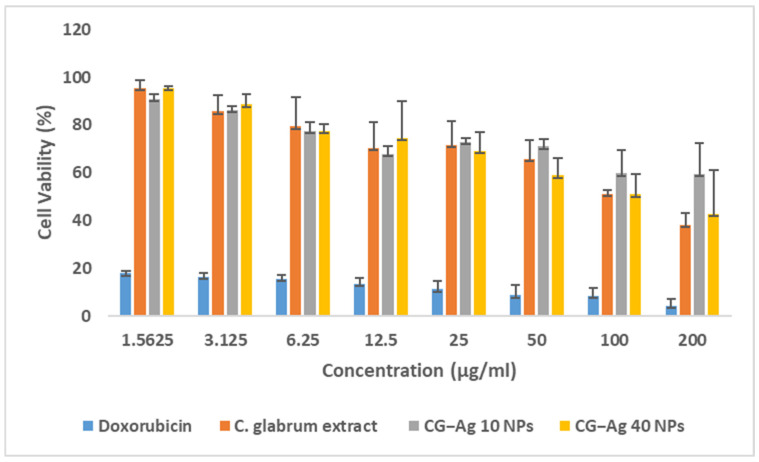
Cell viability percentage for HEK-293 after 24 h of treatment with *C. glabrum* extract, CG-Ag 40 NPs, and CG-Ag 10 NPs at different concentrations (1.5 to 200 µg/mL). Results are reported as mean ± standard error of the mean (SEM).

The 3-(4,5-dimethylthiazol-2-yl)-2,5-diphenyl tetrazolium bromide dye (MTT) assay was used to assess the viability of HEK-293 cells. This assay is based on the ability of viable cells to metabolise the MTT reagent into a purple formazan dye, which is soluble in DMSO. The more intense the purple colour, the more viable the cells. The absorbance of the dye was measured at 550 nm [36,59]. Cell viability decreased as the concentration of the nanoparticles or extract increased. According to Victor Kuete and Thomas Efferth (2015) [60], the strength of a substance’s toxic effect on normal cells can be judged by its IC_50_ value. If the IC_50_ is less than 100 µg/mL, the substance is highly toxic. The toxicity is moderate if it is between 100 and 300 µg/mL. An IC_50_ between 300 and 1000 µg/mL indicates low toxicity. If the IC_50_ is over 1000 µg/mL, the substance is considered not toxic [60]. The results for the CG-Ag NPs and extract are reported in Figure 11.

The *C. glabrum*, CG-Ag 10 NPs, and CG-Ag 40 NPs had IC_50_ values of 139.85, 173.3, and >200 µg/mL, respectively (Table 3). *C. glabrum* extracts displayed low toxicity to THP-1 and Vero cell lines, with cell viability above 50% at 1000 µg/mL. The non-toxic level of *C. glabrum* extract correlates with the lack of toxicity reported due to its traditional uses [53]. The nanoparticles had a high IC_50_ compared to the extracts, indicating the reduced toxicity of the extracts due to the Ag NPs. The overall results show moderate to low cytotoxicity among the nanoformulations. The moderate toxicity shown by the CG-Ag NPs is promising, as toxicity testing is one of the first steps conducted in the development of a drug for any disease.

Similar results were observed in a study of *Elephantorrhiza elephantina* extracts, whereby the synthesised nanoparticles had reduced toxicity against HEK-293 cells compared to the extract alone [61]. *Allium sativum* extract-synthesised Ag NPs showed a low level of toxicity when evaluated against the 3T3 cell line, with only 27.26% cell death at 100 µg/mL [62]. A number of factors, such as the size, morphology, capping agent, cell type, and particle composition, influence the toxicity of Ag nanoparticles [63]. Quercetin showed higher toxicity than its nanosuspension with polyethylene glycol on the human keratinocyte (HaCaT) cell line at 100 µg/mL, with cell viability of 63.8% and 76.9%, respectively [16]. The improved toxicity of phytochemicals when combined with NPs can enable the use of NPs to deliver previously toxic phytochemicals to achieve desired effects.

### 3.10. Investigation of Anti-Diabetic Activity

**Figure 12:**
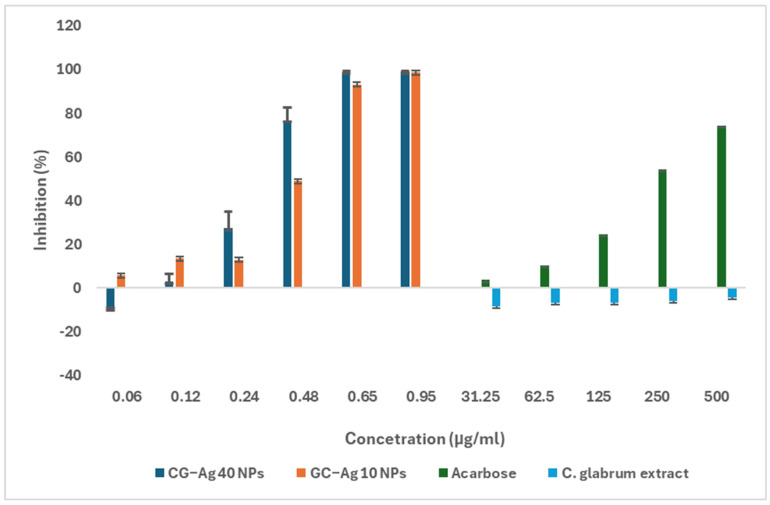
Percentage of α-glucosidase inhibition of *C. glabrum* extract, CG-Ag 40 NPs, and CG-Ag 10 NPs at different concentrations (1.5 to 500 µg/mL). Results are reported as mean ± standard deviation (SD), n = 3.

**Figure 13:**
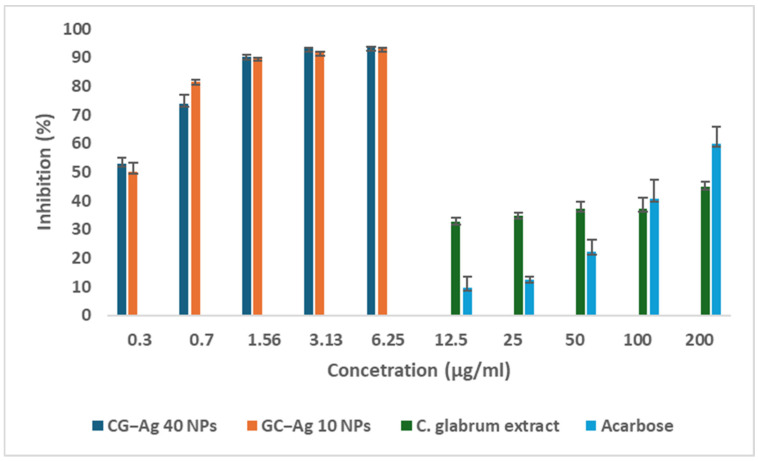
Percentage of α-amylase inhibition of *C. glabrum* extract, CG-Ag 40 NPs, and CG-Ag 10 NPs, at different concentrations (1.5 to 200 µg/mL). Results are reported as mean ± standard deviation (SD), n = 3.

Diabetes is a metabolic disorder associated with hyperglycaemia, oxidative stress, and the excessive production of inflammatory markers, which in turn result in organ damage (mainly the kidneys and liver). One strategy to manage diabetes is to limit the amount of glucose absorbed after eating, which is achieved by inhibiting the actions of α-glucosidase and α-amylase enzymes [64,65]. Figure 12 and Figure 13 show the inhibitory properties of the synthesised CG-Ag NPs for α-glucosidase and α-amylase, respectively. The biosynthesised CG-Ag NPs showed strong α-glucosidase inhibition properties, achieving over 80% inhibition at a concentration of 0.95 µg/mL (Figure 12). The *C. glabrum* extract alone displayed α-glucosidase-enhancing properties. The NPs performed better than the acarbose standard. The acarbose and extract were tested at higher concentrations, as no activity was observed at concentrations similar to those of the NPs. Both acarbose and the CG-Ag NPs showed concentration-dependent inhibitory properties.

The aqueous extract of *C. glabrum* showed mild α-amylase inhibition, as displayed in Figure 13, while the synthesised NPs showed significant inhibition at a lower concentration. This shows the ability of the nanoparticles to act as a delivery vehicle, with the potential to reduce the required concentration to achieve the desired results.

The results are in agreement with previous studies whereby researchers evaluated the ability of Ag NPs to inhibit α-amylase and α-glucosidase. The mechanism behind this is the presence of the *C. glabrum* phytochemical around the Ag NPs. The aqueous root extract of *Martynia annua* inhibited about 78% of α-amylase enzyme at 250 µg/mL [66]. *Asparagus officinalis*-synthesised Ag NPs improved both α-amylase and α-glucosidase, when compared to the crude extract, from 78.6% and 78.7% to 88.8% and 85.3%, respectively. A molecular docking study revealed that Ag NPs interact with various amino acids, such as asparagine/valine residues of α-amylase and glycine residues of α-glucosidase, resulting in inhibition and reduced activity [62].

Biosynthesised silver nanoparticles are garnering considerable attention due to their ability to be developed as anti-diabetic drugs. A comparative analysis (Table 4) of biosynthesised Ag NPs’ ability to inhibit α-amylase and α-glucosidase revealed that the bioactivity of the NPs can be solely influenced by the phytochemicals that reduce and cap the particles. There was no clear pattern indicating the influence of the size and charge.

### 3.11. Hyaluronic Degradation Inhibition Assay

**Figure 14:**
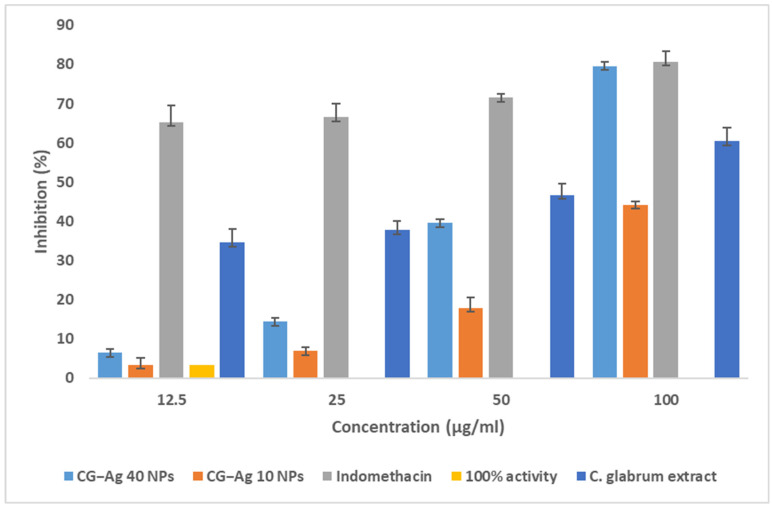
Percentage of hyaluronidase inhibition of *C. glabrum* extract, CG-Ag 40 NPs, and CG-Ag 10 NPs at different concentrations (1.5 to 100 µg/mL). Results are reported as mean ± standard deviation (SD), n = 3.

The ability of the synthesised nanoparticles to inhibit the degradation of hyaluronic acid was evaluated. Hyaluronic acid (HA) degradation by hyaluronidase produces small fragments that act as immunostimulants and pro-inflammatory agents. The CG-Ag 40 NPs showed a remarkable inhibitory rate of 79.6%, compared to the standard drug (indomethacin) with 80.7%, at 100 µg/mL (Figure 14). The *C. glabrum* aqueous extract showed better hyaluronidase enzyme inhibition than the CG-Ag 10/40 NPs at 100 µg/mL, inhibiting degradation by 60.45% and 44. 28%, respectively. *C. glabrum* had an IC_50_ value of 61.95 µg/mL, while the CG-Ag 40 NPs had an IC_50_ value of 63.04 µg/mL. The samples exhibited concentration-dependent bioactivity. The inhibition of hyaluronidase is critical, as there is the upregulation of its activity during chronic inflammation [40]. Therefore, the hyaluronidase inhibition displayed by the CG-Ag NPs shows the potential of *C. glabrum* NPs to be developed as drugs for the management of inflammatory and related disorders.

### 3.12. Nitric Oxide Scavenging

**Figure 15:**
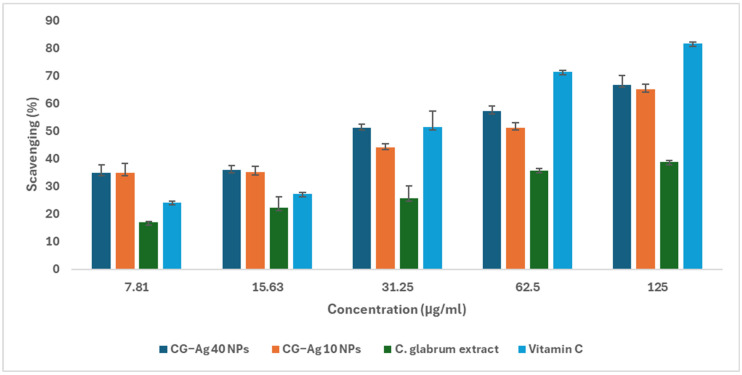
Nitric oxide scavenging activity of *C. glabrum* extract, CG-Ag 40 NPs, and CG-Ag 10 NPs at different concentrations (7.81 to 125 µg/mL). Results are reported as mean ± standard deviation (SD), n = 3.

The ability of the synthesised nanoparticles to scavenge NO was evaluated in terms of the prevention of the formation of nitrite from sodium nitroprusside-generated NO [71]. The nanoparticles showed strong NO scavenging potential (Figure 15). In contrast, *C. glabrum* aqueous extract showed weak nitric oxide scavenging potential (Figure 15). The CG-Ag 10 NPs, CG-Ag 40 NPs, and *C. glabrum* aqueous extract scavenged 65%, 66.7%, and 26.85% of sodium nitroprusside-generated NO, respectively. The CG-Ag 40 NPs (IC_50_ 29.82 µg/mL) scavenged NO better than ascorbic acid (IC_50_: 0.51 µg/mL). The NPs enhanced the NO scavenging ability of *C. glabrum* extract by over two times, from 26% to 66%, highlighting the ability of nanomaterials to improve the efficacy of phytochemicals. *Calophyllum tomentosum* leaf extract-synthesised Ag NPs scavenged 78.46% of NO at 100 µg/mL [72]. *M. peregrina* phytochemically synthesised Ag NPs were good NO scavengers when compared to gallic acid, with over 60% at 80 µg/mL [3].

Both reactive oxygen species (e.g., superoxide radicals) and reactive nitrogen species (e.g., NO) are by-products of cellular metabolism, with the potential to cause cellular harm if not scavenged/neutralised. They cause inflammation by oxidising and modifying genes and proteins, which leads to an inflammatory response and diseases. Therefore, antioxidants are essential in slowing down or inhibiting inflammation [16]. Polyphenols, a common class of phytochemical compounds, are known to act as anti-inflammatory agents by scavenging free radicals, in addition to inhibiting enzymes such as cyclooxygenase and inducible nitric oxide synthase [40]. Phenols were detected during the phytochemical screening of the *C. glabrum* extract.

## 4. Conclusions

CG-Ag NPs capped with biomedically active functional groups from *C. glabrum* were successfully synthesised and were characterised by UV–vis spectra, DLS, EDS, and HR-TEM analysis. The synthesised Ag NPs displayed enhanced biological activity compared to the aqueous extract alone, suggesting dual biological activity from the Ag NPs and the phytochemicals inherited from the extracts. The synthesis process was optimised by varying the AgNO_3_ concentration, time, temperature, and *C. glabrum* extract concentration. The CG-Ag NPs showed good anti-diabetic and anti-inflammatory properties at low concentrations compared to the extract alone by inhibiting α-amylase, α-glucosidase, and hyaluronidase enzymes. The results generated in the study show the potential of CG-Ag NPs to be developed as drugs for the management of diabetes and inflammation. It is worth noting that the next step should be to evaluate the performance of the nanoparticles using cell-based experimental and animal models.

## Figures and Tables

**Table 1:** Phytochemical screening results for *C. glabrum* aqueous extract and GC-Ag NPs.

Phytochemical Constituents	*C. glabrum* Extract	GC-Ag NPs
Terpenoids	+	+
Alkaloids	−	−
Steroids	+	+
Glycosides	+	−
Anthraquinones	−	−
Flavonoids	−	−
Saponins	+	+
Tannins	−	−
Phenols	+	−

Legend: + phytochemical detected; − phytochemical not detected.

**Table 2:** Dynamic light scattering data for the synthesis of CG-Ag NPs.

Sample ID	Size (nm)	Zeta Potential (mV)	Polydispersity Index (PI)
CG-Ag 10 NP	87.12	−25.38	0.3088
CG-Ag 40 NP	148.7	−21.22	0.2687

**Table 3:** IC_50_ values of *C. glabrum* aqueous extract and its biosynthesised nanoparticles.

	*C. glabrum* Extract	CG-Ag 40 NPs	CG-Ag 10 NPs	Doxorubicin
Estimated IC_50_ (µg/mL)	139.85	173.34	>200	<1.56

**Table 4:** Comparative analysis of biosynthesised silver nanoparticles from different plants.

Plant Name	Size (nm)	Zeta Potential (mV)	α-Amylase (Concentration)	α-Glucosidase (Concentration)	Reference
*C. glabrum*	148.7	−21.22	93.32 (6.25 µg/mL)	99.25 (0.95 µg/mL)	In this study
*Azadirachta indica*	ND	ND	73.85% (100 µg/mL)	ND	[32]
*Fagonia cretica*	ND	ND	83.53% (1000 μg/mL)	81.74% (1000 μg/mL)	[64]
*M. annua*	64	−21.6	78.48 (250 µg/mL)	ND	[66]
*Z. officinale* and *O. gratissimum*	ND	ND	78% (50 µg/mL)	80% (50 µg/mL)	[67]
*Stenocereus queretaroensis*	99.5	−32.8	70.1% (10 μg/mL)	99.8% (10 μg/mL)	[68]
*Hebeloma excedens*	190.40	−51.57	50% (7.08 mg/mL)	50% (29.20 mg/mL)	[69]
*Cymodocea serrulata* (R.Br.) Asch. & Magnus	ND	ND	57.31% (125 μg/mL)	54.5% (125 μg/mL)	[70]

No data (ND): analysis was not performed.

## Data Availability

The data presented in this study are available upon request from the corresponding author.

## Abbreviations

The following abbreviations are used in this manuscript

CG	*C. glabrum*
NP	Nanoparticle
Ag	Silver
Ag NPs	Silver nanoparticles
CG-Ag NPs	*Clerodendrum glabrum*–silver nanoparticles
NO	Nitric oxide
MTT	3-(4,5-Dimethylthiazol-2-yl)-2,5-diphenyl tetrazolium bromide
DNS	3,5-Dinitrosalicylic acid
EDTA	Ethylenediaminetetraacetic acid
DMSO	Dimethyl sulfoxide
RPMI	Roswell Park Memorial Institute 1640 medium
DMEM	Dulbecco’s Modified Eagle Medium
PBS	Phosphate-buffered saline
DLS	Dynamic light scattering
HR-TEM	High-resolution transmission electron microscopy
UV–Vis	Ultraviolet–visible spectroscopy
SEM	Standard error of the mean
SD	Standard deviation
